# From rumen to industry

**DOI:** 10.1186/1475-2859-11-121

**Published:** 2012-09-10

**Authors:** Michael Sauer, Hans Marx, Diethard Mattanovich

**Affiliations:** 1 Department of Biotechnology, BOKU-VIBT University of Natural Resources and Life Sciences, Muthgasse 18, Vienna, 1190, Austria; 2 Austrian Centre of Industrial Biotechnology (ACIB GmbH), Muthgasse 11, Vienna, 1190, Austria

**Keywords:** Rumen, Industrial microbiology, Lignocellulose conversion, Microbial community, Microbial ecosystem, Metabolic engineering, Microbial organic acid production, Biofuels

## Abstract

The rumen is one of the most complicated and most fascinating microbial ecosystems in nature. A wide variety of microbial species, including bacteria, fungi and protozoa act together to bioconvert (ligno)cellulosic plant material into compounds, which can be taken up and metabolized by the ruminant. Thus, the rumen perfectly resembles a solution to a current industrial problem: the biorefinery, which aims at the bioconversion of lignocellulosic material into fuels and chemicals. We suggest to intensify the studies of the ruminal microbial ecosystem from an industrial microbiologists point of view in order to make use of this rich source of organisms and enzymes.

## Commentary

**Figure 1:**
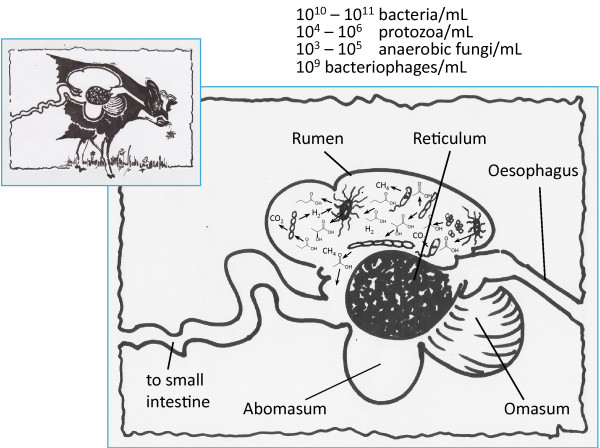
**The rumen is the first chamber in the digestive system of ruminant animals, such as cows.** The digestion of the cellulose rich feed of ruminants occurs through fermentation by microbes. The main substrates for the microbes are structural and non-structural carbohydrates (such as starch, hemicellulose and cellulose), which are hydrolysed into mono- or disaccharides by microbial enzymes. The resulting mono- and disaccharides are assimilated and fermented by the microbial community to volatile fatty acids (VFAs) which are absorbed into the blood stream by the ruminant, and are used as substrates for energy production and biosynthesis. The microorganisms eventually flow out into the omasum and the remainder of the digestive tract. The conditions in the rumen are favourable for the desired microbial community: anaerobic, weakly acidic pH between 5.3 and 6.7, solids content between 12 and 18%. The abomasum is strongly acidic (pH 2 to 4) therefore it acts as a barrier, killing the ruminal microbial flora. Finally, the microbial biomass is digested in the small intestine and smaller molecules (such as amino acids) are taken up by the ruminant.

Due to the great importance of ruminants for our societies and a general interest in the connected microbiolgy the rumen has been studied for a long time. As early as 1950 Robert Hungate compared ruminal to industrial fermentations [1]. However, it is only recently, that the rumen truely comes into the focus of industrial microbiologists. Furthermore, only the recent advent of very high throughput systems biology tools brings us to a point in which we can start to understand this highly complex natural ecosystem (Figure 1).

Right now we see three major points connecting industrial microbiology and rumen biology in an exciting way:

The rumen is a rich source of enzymes for biorefineries

The rumen is a rich source of microbial cell factories

The rumen is a model to study the higher organisational levels of microbial communities, finally leading to a new concept for metabolic engineering

### Enzymes for the lignocellulose based biorefinery

The use of lignocellulosic plant material as carbon source for fuel and chemical production is currently one of the focal points of biotechnology [2]. However, the plant cell wall evolved for stability and robustness and not as a carbon storage compound. Consequently, the enzymatic deconstruction of the plant cell wall poses significant challenges due to its chemical and physical complexity. Despite major investments in research and significant advances in technology, this remains one of the critical points for the establishment of economic biorefineries. Generally, the plant material has to be subjected to a pretreatment followed by enzymatic hydrolysis to obtain a microbially accessible carbon source. A wide variety of bacteria and fungi serving as decomposer in the natural carbon cycle secrete industrially useful cellulolytic enzymes [3].

Shifting our view to ruminants the situation is quite similar. Rumination is the pretreatment of the biomass to render it enzymatically accessible. After rumination the biomass is degraded in the rumen by the complex microbial community and converted into compounds, which are useful for the ruminant. This resembles a modern biorefinery [4]. Also in this case the involved enzymes are of bacterial as well as fungal origin, whereby the bacteria seem to be more important [5].

A recent example published in this journal highlights the suitablity of the rumen as a playground for the industrial microbiologist: Zheng et al. [6] set out to engineer *E. coli* for succinate production from hemicelluloses. In their search for xylanolytic enzymes 3 out of 7 possible enzyme sources were ruminal bacteria. In fact, the combination of one enzyme from *Fibrobacter succinogenes*, a ruminal bacterium, and another enzyme from *Fusarium graminearum*, a plant pathogen fungus turned out to have optimal enzymatic properties for xylan hydrolysis.

### Microbial cell factories found in the rumen

The rumen community with its peculiar interconnections comprises a plethora of microorganisms, which produce chemicals of interest. Here, nature provides us with a wealth of production hosts, which are only scarcely known up to now. Organic acids are the main chemicals used as currencies which are converted among the microorganisms and the microbial community with their host. Organic acid producers are therefore among the best studied ruminal cell factories so far. A prominent example is the microbial production of succinic acid. In fact, succinate is an important metabolic intermediate in the rumen. Several bacteria obtain energy by decarboxylating succinate to propionate, which in turn serves as a nutrient for the ruminant [7]. Among the identified ruminal succinic acid producers are *Anaerobiospirillum succiniproducensActinobacillus succinogenes*, and *Mannheimia succiniproducens*, just to name a few [8]. The importance of the characteristics of the natural environment for the production process has recently been highlighted in this journal for *A. succinogenes* by Zou et al. [9]: The main fraction of the gaseous phase in the rumen is CO_2_. CO_2_ is also a substrate for phosphoenolpyruvate carboxykinase, a key enzyme for succinic acid biosynthesis. Thus, a significant influence of the CO_2_ provision on succinic acid production appears plausible. Zuo et al. [9] could show that not only gaseous CO_2_ but also the presence of carbonates, such as MgCO_3_ are beneficial for succinic acid production. The key message here is that the industrial microbiologist should never forget, where his favourite microbial cell factory comes from, when designing the bioprocess.

A second example for an industrially interesting ruminal organic acid producer is *Megsphaera elsdenii*. *M. elsdenii* readily accumulates short chain aliphatic organic acids such as butyric, valeric or caproic acids when cultured under appropriate conditions [10]. Particularly, caproic (hexanoic) acid production is of interest as it could open a sustainable production route for Nylon. Recently, we obtained the genome sequence of this organism [11]. Deducing the metabolic pathway for hexanoic acid production we speculate that the production of hexanoic acid serves as an electron sink, enabling *M. elsdenii* to get rid of reduction equivalents, growing on glucose. Again, one has to consider the anaerobic environment (no oxygen as electron acceptor) and the profound competition for sugars as carbon sources in the rumen. So it appears plausible that this organism seeks to take up as much carbon source as quickly as possible - even on the expense of carbon for electron dissipation. Apart from the peculiar metabolic activities, *M. elsdenii* is an interesting candidate as microbial cell factory due to a striking resistance against short chain fatty acids – again pointing to the natural environment, where it thrives.

### Microbial ecosystems biology as starting point for metabolic engineering

A microbial ecosystem can be defined as a system that consists of all the microorganisms that live in a certain area or niche and that function together in the context of the other biotic and abiotic factors of that niche [12].

The rumen is a fine example of such an ecosystem, functioning together with the ruminant, that provides the bioreactor (the rumen) and the pretreated carbon source (plant material), and receives in turn carbon and energy in a suitable chemical form. Overall the system can be viewed as one “super metabolism” which is distributed over a variety of organisms and species. The concept here is that particular metabolic pathways are distributed over different organsims. A changing environment, will lead to a change of that metabolism – on one hand by classical mechanisms, such as control of enzymatic activities or transcriptional control. On the other hand a change in community composition (relative numbers or even addition or omission of species) adds another possible layer of regulation.

As a concept this is quite appealing for a biorefinery. Instead of constructing one organism, which converts the carbon source directly into a useful product, one can think of the construction of microbial communities. Some strains enable the decomposition/deploymerisation of different feed stocks. Some strains detoxify the feed stock. Other microorganisms produce the chemicals of interest. Depending on the feed stock and depending on the desired product, the community can be composed in a different way. In other words: metabolic pathways are confined to individual species. By combining the right species - that is the right pathways - the metabolism can be changed - without cloning.

However, at the time being we are far from a detailed understanding of the ongoing in the rumen. While a wealth of new systems biological methods is becoming available [13] we still need to refine our conceptual basis to translate species- and community-data into a framework accounting for the interactions between metabolic networks of species in community enviroments. The rational de-novo construction of an artificial microbial ecosystem is one further step ahead.

However, the key message here is that understanding nature opens our view to exploit the wealth of possibilites in the future.

## Authors’ contributions

All authors have read and approved the final version of the commentary.
